# Interpersonal Perfectionism and Perceived Control’s Influence on the Continuity of Sports Practice in Adolescents

**DOI:** 10.3390/ijerph22010070

**Published:** 2025-01-07

**Authors:** H. Antonio Pineda-Espejel

**Affiliations:** Facultad de Deportes, Universidad Autónoma de Baja California, Mexicali 21100, Mexico; antonio.pineda@uabc.edu.mx

**Keywords:** perceived control, descriptive norm, behavioral intention, public health

## Abstract

Based on the theory of planned behavior, the objective was to test a theoretical model that explains the intention to continue practicing sports among adolescents currently involved in sports practice in Mexicali based on factors that generate perceived social pressure to be perfect (perceived descriptive norm) and that lead to internal factors of perceived control (perceived competence, general self-concept, and enjoyment). A battery of questionnaires that measured the study variables was applied to 195 adolescent athletes of both sexes. The causal model with observed variables rejected part of the hypothesis since the athletes’ perception that their parents impose high performance expectations on them and that they criticize them when these expectations are not achieved was not associated with the athletes’ perceived competence. Meanwhile, perceived coach pressure was positively associated with perceived competence, suggesting that it may be adaptive. This also suggests that perceived competence favors the intention to continue practicing sports both directly and indirectly through self-concept and enjoyment. In conclusion, boys involved in sports will have the intention to continue practicing if they perceive that their closest social references demand that they be perfect in the sport and when they perceive themselves to be able to do it effectively. Meanwhile, girls will have the intention to continue in sports practice if, in addition, they have a better general self-concept and enjoy the activity. Therefore, an important task for coaches, in order to encourage the continuation of sport in adolescents, is to promote the perception of qualities, skills and abilities toward sport.

## 1. Introduction

Sports practice is beneficial for the physical and mental health of children and adolescents [1], but half of children and adolescents who begin a sports activity tend to drop out within the first six months [2]. Therefore, it is important that boys and girls intend to maintain regular sports practice since, even if a behavior is performed many times, it is not guaranteed that it has become habitual [3].

Within the study of the intention to engage in sports, there is research about the influence of coaches and/or parents on intention, which has been conducted from the perspective of autonomy support [4,5]. However, studies that examine the role of interpersonal perfectionism as a descriptive norm are scarce; therefore, in our opinion, the study of the consequences of interpersonal perfectionism is limited. In summary, the literature highlights the need to expand information regarding the variables that most favorably influence the intention to engage in sports during adolescence.

### 1.1. Behavioral Intention

The Theory of Planned Behavior (TPB) [6,7] postulates that (1) the intention to perform a behavior is the immediate antecedent of the behavior; (2) intention, in turn, is determined by the attitude toward the behavior, the subjective norm, and perceived behavioral control; (3) these determinants are themselves a function of behavioral factors, normative beliefs, and control beliefs; and (4) behavioral, normative, and control beliefs can vary depending on a wide range of background factors, such as cultural, personal, and situational factors [3]. Thus, this theory allows consistent prediction of behavioral intentions based on attitudes, perceived social norms, and perceived behavioral control [8]. The importance is that the stronger the intention, the more likely the behavior will follow [9].

For attitudes, these generally do not provide a good basis for predicting and explaining specific behaviors over time [3]. Regarding subjective social norms, these are perceptions of social pressure due to considerations related to the likely approval or disapproval of a behavior by friends, family, and coworkers, among others, including descriptive or behavioral norms, that is, perceptions of others’ behaviors [10]. Perceived behavioral control is a person’s perception of his or her ability to carry out a certain behavior. Unlike the other two types of perception, only behavioral control can directly or indirectly predict behavioral intentions [3].

### 1.2. Perceptions of Social Pressure

Social agents play an important role in young people [11]. In sports, there are three main sources of interpersonal influence as an athlete progresses in his or her sporting career: family, coach, and teammates [12].

Parents play an especially important role during childhood and adolescence [13]. In the sports context, parental involvement and parents’ relationships with their children are essential [14], as they are primarily the ones who provide children with their first opportunity to participate in sports, and their influence on the decision to continue or quit is significant [15]. Furthermore, in this context, coaches’ behavior can have a crucial impact on athletes because they design practice sessions, give recognition [16], then could influence the intention to continue practicing sports among individual athletes or sports teams with which they work.

The sports context is characterized by the value placed on competition, social comparison, an emphasis on winning, and public recognition of displayed skills [16]. This type of context can create social environments considered to be more or less perfectionistic [17]. Therefore, regarding the perceptions of interpersonal behaviors in sports, it is possible to rely on multidimensional perfectionism theories [18], which recognize both personal and interpersonal aspects.

Among interpersonal aspects, there is socially prescribed perfectionism, which is the belief that others have excessively high standards for oneself, and that acceptance or approval from others is conditional on fully meeting those standards [18]; this is also called perfectionism pressure (PP). This form of perfectionism is considered maladaptive, as it shows a positive relationship with negative outcomes such as negative affect and a negative relationship with outcomes such as positive affect and self-esteem [19,20].

Flett et al. [21] suggested the perfectionism parental pressure facet (PPP), which combines the perception that parents place high expectations on their children and critically evaluate their performance if they fail to meet those expectations. In the sports field, Dunn et al. [22] added the perfectionism coach pressure facet (PCP) given that a coach is a determining authority figure in the experiences of athletes.

### 1.3. Perceived Control

Ajzen [23] analyzed several internal factors, such as skills, abilities, and emotions, that can influence the degree of control that a person has over a given behavior. Therefore, an alternative measure of perceived control associated with ability is perceived competence, which is the perception of oneself that he or she can effectively handle situations associated with sports. Those with high perceived competence develop a high self-concept [8]. This self-concept refers to cognitive aspects and is defined as the knowledge and beliefs that a subject has about himself or herself in all the dimensions and aspects that shape him or her as a person [24].

Moreno et al. [25] found that among secondary school physical education students, the intention to be physically active was positively predicted by perceived competence and, to a lesser extent, self-esteem. Similarly, in the study by Cuevas et al. [26], one of the variables that most strongly predicted the intention to be physically active was perceived competence (understood as a component of self-concept). Outside the sports context, Hassandra et al. [27] considered the role of self-concept, indicating that self-concept can significantly improve the prediction of intentions [8].

For emotions, positive states are also distinguished, which can be considered an experiential element of attitudes toward behaviors [3,28]. Among the positive states is enjoyment, which is defined as a positive affective state or a positive attitudinal response toward the sport experience, reflecting feelings such as pleasure and fun [29]. There is evidence that in school-aged populations, one of the main reasons for engaging in sports is the feeling of enjoyment [30].

Perhaps the most frequently mentioned yet seemingly overlooked bias factors in the TPB are positive states and emotions. It is often suggested that affective states can directly influence behavior, and that this possibility is not sufficiently considered in the TPB [28]. However, it is also theorized that emotions can have a strong indirect impact on intentions [3].

### 1.4. Present Study

It has been suggested that the constructs contained in the TPB may not be sufficient to fully explain people’s intentions and actions [31], as intentions may be determined not only by attitudes, norms, and perceived control but also by one or more additional variables, suggesting that one or more predictors can be added [28].

The possibility of specifying, identifying, and estimating an alternative model within the same theoretical framework, including additional predictor variables, can improve the prediction of intentions [3]. Therefore, this study added family (PPP), sport (PPE), and personal (perceived competence, self-concept, and enjoyment) psychological variables that may facilitate or interfere with sports participation. An experiential element (i.e., enjoyment) was added because much of the research conducted within the framework of the TPB has devoted little attention to the role of affective states in the prediction of intentions [3]. Based on Ajzen [28], these additions were defined at a level compatible with the behavior in question, conceived as causal factors that determine intention, some of which are conceptually independent of the existing predictors of the theory, with the aim to improve the prediction of intentions.

For the above reasons, the objective of this study was to test a sequential theoretical model that explains the intention to continue practicing sports among adolescents engaged in sports in Mexicali based on factors that generate perceived social pressure to be perfect from parents and coaches (perceived descriptive norm), which lead to internal factors of perceived control (perceived competence, general self-concept, and enjoyment) regarding sports performance. Following Ajzen et al. [3], this study considered the demographic variable sex to be a control variable.

**Figure 1:**
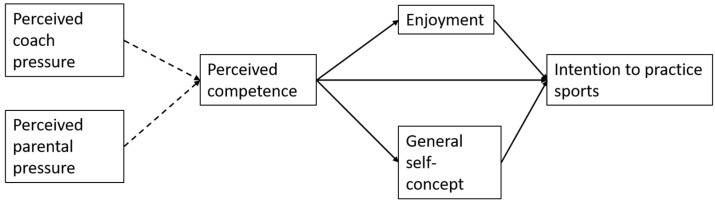
Hypothesized model. Dashed lines indicate a negative association, and solid lines indicate a positive association.

Guided by theory and previous research and given that the dimension of interpersonal perfectionism is considered maladaptive, in this study, it was expected that (1) the pressure for perfection would be negatively associated with perceived competence; (2) perceived control (perceived competence, self-concept and enjoyment) would be positively associated with consequences of the intention to practice sports; and (3) perceived competence would directly influence intention (Figure 1).

## 2. Method

This study followed an empirical research design, applying an associative strategy of a cross-sectional explanatory type with observed variables [32].

### 2.1. Sample

Using non-probabilistic convenience sampling, 195 athletes participated (87 boys; 108 girls), who were aged between 14 and 15 years (M = 14.08 years; SD = 0.40). The sample size was determined based on the recommendation of 5–10 subjects per parameter to be estimated, ensuring the minimum required according to the inverse square root method [33], with a significance level of 5% and a minimum path coefficient of 0.2 [34]. A power calculation of the sample using G*Power 3.1 supported the sufficiency of the sample to reject the null hypothesis with an effect size of f^2^ = 0.35 at a confidence level of 99% and power of 95%. The sample was collected from both municipal sports schools and sports clubs in Mexicali, Mexico. The athletes competed at the municipal level in sports such as American football (19%), athletics (19%), soccer (25%), swimming (17%), and volleyball (20%) and reported a time dedicated to sports practice of between 1 and 5 years. Sample characteristics by sport are presented in Table 1.

### 2.2. Instruments

To measure interpersonal perfectionism, the parental pressure subscale of the Spanish version of the Multidimensional Sport Perfectionism Scale–2 (S-MPS-2) [35] was used. It consists of nine items (e.g., “My parents expect excellence from me in my sport”) answered on a five-point Likert scale ranging from strongly disagree (1) to strongly agree (5).

Additionally, the coach pressure subscale of the Spanish version of the Multidimensional Inventory of Perfectionism in Sport (MIPS) [36] was used. It consists of six items (e.g., “My coach expects me to be perfect”) that follow the initial phrase “During my training sessions…”. These are answered on a six-point Likert scale ranging from never (1) to always (6).

The Spanish version of the Intrinsic Motivation Inventory (IMI) [37] was used to measure perceived competence and enjoyment. Five items assess competence perception (e.g., “I think I am pretty good at my sport”), and five items assess interest/enjoyment (e.g., “Playing my sport is fun”). Responses are given on a five-point Likert scale ranging from totally disagree (1) to totally agree (5).

To measure the global component of self-concept, the General Self-Concept subscale of the Spanish version of the Self-Description Questionnaire for Adolescence (SDQ-II) [38] was used. This global component integrates self-perceptions of self-worth, self-confidence, and self-satisfaction. It consists of 10 items (e.g., “In general, I have a lot of confidence in myself”) preceded by the phrase “In relation to yourself…”, where individuals rate themselves as effective, capable, proud, and satisfied with who they are. Responses are recorded on a six-point Likert scale ranging from false (1) to true (6).

To measure the intention to practice sports at the present time, the measurement of the Intention to be Physically Active (MIFA) [25] was used. It is composed of five items (e.g., “I am interested in developing my physical fitness through sports”) preceded by the phrase “Regarding your intention to continue practicing your sport in the coming months…”. These are answered on a five-point Likert scale ranging from totally disagree (1) to totally agree (5).

In addition, to describe the sample, sociodemographic data such as age, sex, sport currently practiced, length of practice, and level of competition were collected.

### 2.3. Procedure

The study met the ethical guidelines proposed by the American Psychological Association (APA) and received ethical approval from the University (UABC-149/3/C/9/23). First contact was made with coaches to explain the study’s objective and ask for permission to provide the questionnaire book. Informed consent was obtained from the parents of all participants. The questionnaires were completed at the sports venues during the sports preparation period, at the first weekly training session, and before starting the warm-up; the trainer was not present, but the researcher was present in order to resolve any doubts that might arise in the understanding of the items. The voluntary participation, anonymity, and confidentiality of the data received was requested. The subjects had 30 min to answer the questionnaires.

### 2.4. Statistical Analysis

First, we confirmed that there were no missing values or outliers. The measurement models were tested with a confirmatory factor analysis (CFA) for each instrument, as well as internal consistency analysis by calculating Cronbach’s alpha coefficient. Subsequently, univariate normality and descriptive analyses of the variables were performed, followed by bivariate correlation analysis between the variables, using SPSS version 23. Then, to achieve the objective of the study, a causal model with observed variables (path analysis), controlled by the sex variable, was tested using the maximum likelihood estimation method. Both the CFAs and the path analysis were tested with the program Mplus version 7.3.

To evaluate the fit between the theoretical model proposed and the data matrix collected, fit indices such as the ratio of χ^2^ and degrees of freedom (*df*) were used, where values between 2 and 3 represent an acceptable fit [39]. In addition, the RMSEA and its 90% confidence interval (90% CI), the SRMR, and the TLI and CFI incremental indices were evaluated. According to various approximations, the cut-off points (in parentheses) for acceptable model fit (CFI > 0.90, TLI > 0.90, SRMR < 0.10, RMSEA < 0.08) [40] and for good model fit (CFI > 0.95, TLI > 0.95, SRMR < 0.08, RMSEA < 0.05) [40] were followed in this study.

A multi-group invariance analysis was performed to assess group differences in the extent of paths between boys and girls. A model with all paths constrained to be equal across groups (constrained model) was compared to a configural model with all paths freely estimated (free model). The results for each invariance test were explained by changes in the CFI (ΔCFI) and RMSEA (ΔRMSEA) values, where differences less than 0.01 for the first value and 0.015 for the second value indicated irrelevant practical differences [41].

Lastly, mediation effects within the model were tested with the bootstrapping method (1000 samples) and the 95% confidence interval (95% CI) to determine the standardized values and significance levels of indirect effects. Indirect effects were considered significant if the 95% CI did not pass through zero [42].

## 3. Results

### 3.1. Preliminary Analyses

Evidence of validity was offered based on the internal structure of the instruments used, where the CFAs for each instrument showed adequate data fits (Table 2). Cronbach’s alpha reliability coefficients for the scales were satisfactory, exceeding the criterion of 0.70. According to the descriptive analysis of the present work, the kurtosis and skewness values for the variables ranged between −1 and 1. Table 3 shows the means, standard deviations, and bi-variate correlations of the variables that comprised the model. The mean scores indicated that the participants generally scored around the middle of the scales for overall self-concept and perfection pressure and showed high levels of sports practice intention, enjoyment, and perceived competence. The correlation matrix confirmed the assumption of linearity between the dependent and independent variables. Regarding interpersonal perfectionism, perfection pressure was positively related to perceived competence, while general self-concept, enjoyment, and perceived competence were positively related to the intention to practice sports.

### 3.2. Primary Analysis

In the main analysis, compliance with the assumptions of the model was confirmed as follows: (1) non-co-linearity, since the diagnosis of co-linearity showed that the tolerance statistics were above 0.01 (between 0.53 and 0.75), the variance inflation factors were less than 10 (between 1.33 and 2.08), and the condition index was equal to or less than 15 for each dimension (between 6.25 and 15.09); (2) independence of the residuals, since the Durbin–Watson value was 1.87; (3) homoscedasticity of residuals, since the variance of the residuals was uniform throughout the range of predicted values; and (4) normality of the residuals, since the fit of the residuals to the normal curve was verified.

**Figure 2:**
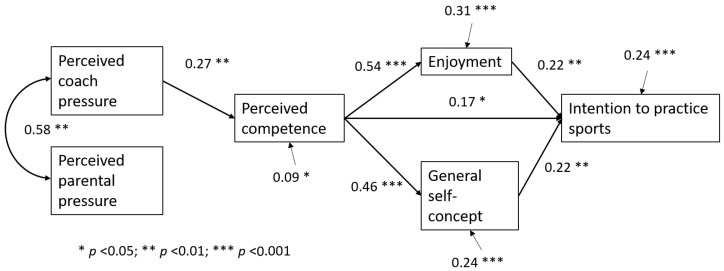
Standardized solution of the model tested and controlled for sex. Only significant effects are shown. Explained variances are presented with the small arrows.

The results of the path analysis, controlled for sex, indicated that the model provided a good representation of reality: χ^2^ = 11.67, *df* = 5, *p* < 0.05, CFI = 0.96, TLI = 0.90, RMSEA = 0.08, 90% CI [0.06, 0.09], and SRMR = 0.04. Figure 2 shows the significant relationships in the path analysis, including the PCP being positively and significantly associated with perceived competence (β = 0.27; *p* < 0.01) and perceived competence being positively and significantly correlated with enjoyment, general self-concept, and sports practice intention (β = 0.54; *p* < 0.001; β = 0.46; *p* < 0.001; β = 0.17; *p* < 0.05, respectively). At the same time, both enjoyment and self-concept were positively and significantly associated with the intention to practice sports (β = 0.22; *p* < 0.01, in both cases). This model explained 24% of the variance of the outcome variable.

**Figure 3:**
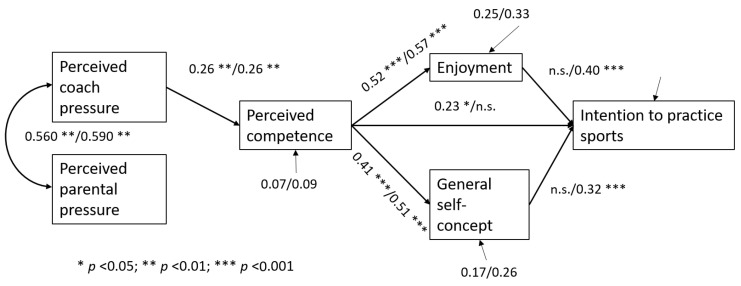
Standardized path coefficients of the model of the two groups (boys/girls). R^2^ values are noted with an arrow. Note. n.s.: no significant.

The multigroup analysis comparing the model between adolescent boys and girls indicated that, initially, the theoretical model had an adequate fit in both subsamples, so it was appropriately specified, and a proper solution was obtained. The model was then found to be equivalent across the two groups, from configural invariance (factor patterns were equal) to structural weights (structural relationships among observed variables were equal), since the differences in the fit indices were trivial (see Table 4). The regression coefficients are presented in Figure 3, reflecting that the relationships between the variables were the same; however, in boys, perceived competence had a direct effect on intention (β = 0.23, *p* < 0.01), while in girls, intention was associated with enjoyment (β = 0.40, *p* < 0.001) and general self-concept (β = 0.32, *p* < 0.001).

### 3.3. Indirect Effects

Based on Figure 2 and the multi-group analysis results, the calculation of mediation effects of the enjoyment and self-concept variables indicated that both variables mediate the relationship between perceived competence and girls’ intention to continue practicing sports since the 95% confidence interval of the indirect effect did not include the value zero (Table 5).

## 4. Discussion

This study was carried out with the aim of testing a theoretical model that explains the intention to continue practicing sports among adolescents who are immersed in sports practice in Mexicali based on factors that generate perceived social pressure to be perfect (perceived descriptive norm) and that lead to internal factors of perceived control (perceived competence, general self-concept, and enjoyment).

With respect to the hypothesized model, the first part of the sequence (the PCP would have a negative effect on perceived competence) was rejected, as the athletes’ perception that their coaches expect them to be perfect and criticize them when they fail to achieve high standards was positively associated with the athletes feeling competent in their sports rather than negatively affecting it. Perceived parental pressure was not associated with the athletes’ own perception of competence.

The high expectations imposed by coaches may be experienced by adolescents as a useful impetus for acquiring sports skills or pursuing excellence. However, adolescent athletes may develop emotional dependencies as a consequence of looking up to or seeing their coaches as role models and then viewing the pressure for perfection as favorable, which helps them avoid rejection, reinforce conditional self-esteem for achievement, and obtain contextual approval [43].

Contrarily, athletes’ perception that their parents expect them to be perfect in sports and criticize them when they fail to meet their high expectations does not affect their own perception of competence. It appears that parental influence is important in the general domain, whereas coaches are important in the sports context [44], suggesting complexity in interpretations of parental involvement [45]. This may be because as adolescent athletes become more involved in their sports and coaches become a primary influence, while parents take a back seat and play a less direct role in their children’s practice. As a result, young athletes may experience lower sports-related expectations from their parents [46], as they are less involved in their children’s sport activities than in other aspects of life in general. Another possible explanation is that as children become older, the perception of high expectations from their parents or the importance that they place on this perception decreases [44]. Since no studies were found that included all of these variables or similar studies, it is not possible to support these results with previous evidence.

Although the meta-analysis by Madigan et al. [44] demonstrated that both parents and coaches play important roles within sport through perfectionism, in the present study, only the high standards imposed by coaches and their critical evaluation after failing to achieve those standards contributed to desirable consequences such as increasing one’s own perceived competence in sports. This may be because coaches spend much of their time with young athletes during training, or because they are seen as a close and meaningful source of information.

In addition, regarding socially prescribed perfectionism, Campbell and Di Paula [47] inspected the items that constitute this factor on the Multidimensional Perfectionism Scale [18] and found two factors, including one that they called high standards of others and another called conditional acceptance. Of these two subscales, only conditional acceptance has shown negative relationships with outcomes such as self-esteem and positive affect and a positive relationship with negative affect. Thus, high standards of others may capture less negative or even ambivalent aspects [48].

Such subdimensions can also be seen in the instruments used here, so our results could be due to the combined effects of PP (high standards of others + conditional acceptance), or the effect of one aspect may cancel out the effect of the other. Nevertheless, the evidence for the existence of the two subscales is still rather weak [48] and remains untested in the adolescent sports context.

Thus, it is supported that PP can function as a descriptive norm within the TPB, which reinforces that perfectionism manifests differently in different life domains [49]; the interpersonal aspects of perfectionism in sports have different effects on athletes’ experiences, being able to be positive from coaches and without influence from parents.

The next part of the model sequence confirms that in girls, perceived competence favors having a better idea of themselves (general self-concept) and greater possibilities of enjoying sports practice, which is consistent with some studies [25,50,51]. This is because perceived competence may be a cognitive component of self-concept, and then the strength of the latter is based on beliefs in one’s own abilities, supporting that those with high perceived competence develop a high self-concept [8].

Ajzen and Schmidt [9] mention that the reason for continuing or stopping a certain activity is related to a high or low perception of behavioral control. In this work, the final part of the model confirmed that in boys, the intention to continue sports practice is related to perceived competence. This supports perceived competence as a good predictor of intention [3]. It corroborates that adolescents tend to continue sports practice when they feel competent and enjoy participation [51,52].

The associations of perceived competence—intention (in boys) and general self-concept—intention (in girls) coincide with data from other studies, such as the study of Di Battista et al. [53] or the studies of Moreno et al. [25] and Cuevas et al. [26], where one of the variables that was most strongly associated with intention was perceived competence. These findings also agree with studies such as Grao-Cruces et al. [54], who found that in high school students, a more positive self-concept leads to greater intentionality to be physically active. Thus, self-concept improves the prediction of intention [8], perhaps because sports practice improves physical fitness and physical ability, which are associated with self-concept and perceived competence, thus favoring the intention to continue.

The enjoyment–intention relationship (in girls) supports that positive states can serve as internal factors influencing control beliefs [28], with affective state being an experiential element of attitudes toward behaviors [3], which has a strong direct impact on intentions [28]. This coincides to some extent with the study of Teixeira et al. [55], where enjoyment stood out as the most relevant predictor of intention to persist in swimming training. Accordingly, enjoyment is associated with the intention to continue sports practice [56] because positive affective states are related to developing plans and goals in the future [57]. This is important as Crane and Temple [58] demonstrated that enjoyment and intention are two important variables for future participation in sports.

This study has theoretical contributions. First, it intended to delve into some psychological variables in the intention process on the personal level of an athlete and in his/her sporting context. Second, it included the influence of a combination of social factors, beyond parents, as a descriptive norm, which has not been previously studied, and it took into account experiential elements of affective states in the explanation of intention, which is seemingly ignored in the TPB. The variables mentioned seem to extend beyond the TPB, but they could be accommodated to broaden and enrich our understanding of the intention to continue sports practice and improve the prediction of intentions.

## 5. Practical Implications

This study helps to identify key points or more appropriate objectives for specific strategies that favor the intention to continue practicing sports in adolescents immersed in sports practice in Mexicali. It is important that from an early age, they adopt active lifestyle habits, which are prolonged over time [59], that last into adulthood [60].

By examining a sample of adolescents involved in habitual sports practice, evidence is offered that helps support a shift from a state of action to a state of behavior maintenance over time [61], a phase that is related to intention states and current behavior [9]. However, we must clarify that intentions tend to change over time and across circumstances.

From the results, an interaction that promotes mutual benefits for athletes and coaches is suggested. That is, coaches can expect high standards and combine them with a variety of tasks and strategies during training sessions that are intended to promote enjoyment and, subsequently, greater persistence. In order to encourage adolescents to stay in sports, coaches could offer positive feedback, inform the athletes or team about the abilities available to them to achieve a goal, establish realistic expectations together with the athletes, offer sufficient time for learning or perfecting tasks, and encourage participation in training tasks. Additionally, with girls, they could diversify the tasks.

## 6. Limitations

This study also has limitations, such as the fact that all measures were collected by self-report and from the extraction of subscales from instruments. Furthermore, the measures did not consider perfectionist profiles given that only an interpersonal aspect of perfectionism was included. The unique effects of conditioned approval and the high standards of others were also not considered given that such a structure has not been tested in the sports context. The cross-sectional design does not allow us to extract causality effects.

Furthermore, the size and characteristics of the sample, which was representative of a special population in a very restricted age range, do not allow generalization of the results, and the findings may not be sufficient to fully explain the intentions of athletes; thus, it is suggested to interpret them with caution, since the variables may behave differently across different countries or cultures. The influence of peers, who may have a significant role in the complete understanding of social factors impacting adolescent sports participation, was also not considered.

Consequently, future studies with different and larger sample sizes are needed to expand these findings or to consider a structural equation model to estimate the error term. Cross-cultural and longitudinal work is also needed to determine whether these effects occur across cultures or over time.

## 7. Conclusions

The results suggest that the intention to continue sport practice among these adolescents immersed in sports in Mexicali is facilitated by internal factors of perceived control (perceived competence, general self-concept, and enjoyment), and that perceived competence is increased by the PCP, while the PPP is irrelevant and does not contribute significantly.

Boys interested in sports will have the intention to continue practicing if they perceive that their closest social references demand that they be perfect in a sport and when they perceive themselves to be able to execute this effectively. Meanwhile, girls will have the intention to continue in sports practice if, in addition, they have a better general self-concept and enjoy the activity.

## Figures and Tables

**Table 1:** Study sample characteristics.

Sport	Age Mean (SD)	Boys (n)	Girls (n)
American football	14.72 (0.40)	25	0
Soccer football	14.02 (0.35)	34	10
Swimming	14.06 (0.48)	16	16
Volleyball	14.26 (0.81)	0	54
Athletics	14.08 (0.06)	12	28

**Table 2:** Fit indices of the measurement scales.

Instrument	χ^2^ (Sig.)	*df*	CFI	TLI	RMSEA (90% CI)	SRMR
PPP subscale of S-MPS-2	64.19 (*p* < 0.001)	24	0.93	0.90	0.08 (0.06–1.00)	0.05
PCP subscale of MIPS	39.65 (*p* < 0.001)	17	0.96	0.94	0.05 (0.04–0.09)	0.03
Perceived competence and enjoyment subscales of IMI	43.92 (*p* < 0.001)	13	0.97	0.95	0.07 (0.01–0.09)	0.03
General self-concept subscale of SDQ-II	14.42 (*p* < 0.001)	5	0.96	0.97	0.04 (0.03–0.05)	0.03
MIFA	11.1 (*p* < 0.001)	5	0.97	0.95	0.07 (0.01–0.09)	0.03

**Table 3:** Descriptive statistics, reliability (diagonal), and Pearson’s correlation matrix between the study variables.

	PCP	PPP	Perceived Competence	Enjoyment	General Self-Concept	Intention to Practice Sports
PCP	0.94					
PPP	0.35 **	0.94				
Perceived competence	0.23 **	0.21 **	0.90			
Enjoyment	0.06	0.06	0.54 **	0.95		
General self-concept	0.10	0.34 **	0.46 **	−0.01	0.95	
Intention to practice sports	0.26 **	0.13	0.40 **	0.39 **	0.37 **	0.90
Range	1–6	1–5	1–5	1–5	1–6	1–5
*M*	3.10	2.48	3.90	4.65	4.30	4.28
*SD*	1.23	0.094	0.94	0.52	1.07	0.83

** *p* < 0.01; PCP: perceived coach pressure; PPP: perceived parental pressure.

**Table 4:** Fit indices for the multi-group invariance test.

Model	χ^2^ (*df*)	*p*	RMSEA	ΔRMSEA	CFI	ΔCFI
Boys	14.98 (5)	0.035	0.08		0.95	
Girls	13.99 (5)	0.020	0.07		0.94	
Unconstrained	19.22 (14)		0.044		0.981	
Structural weights	26.95 (21)	0.35	0.038	0.006	0.978	0.003
Structural covariances	27.85 (24)	0.82	0.029	0.015	0.986	0.005
Structural residuals	36.44 (28)	0.07	0.040	0.004	0.969	0.012

**Table 5:** Indirect mediation effects.

Precedent/Mediator	Outcome	a	SE	b	SE	Product Term ab	95% CI Upper	95% CI Lower
Perceived competence/enjoyment	Intention to practice sports	0.54	0.05	0.22	0.07	0.12	0.02	0.22
Perceived competence/self-concept	Intention to practice sports	0.46	0.05	0.22	0.07	0.10	0.01	0.19

SE = standard error.

## Data Availability

The data presented in this study are available on request from the corresponding author.
